# Letter from Weisbaden, Germany

**Published:** 1885-01

**Authors:** 


LETTER FROM WIESBADEN, GERMANY.

For a people so fond of beer the Germans are certainly very fond of water. Possibly it is owing to their great fondness for beer that they are compelled at last to cultivate a taste for water. Livers and kidneys embarrassed by beer are glad enough to find relief through milk and water and grapes. No matter what the cause may be, the fact stands that the German springs are the most largely patronized of all springs, and that the great majority of these patrons are Germans. The Wiesbaden Springs are among the most, if not *the* most famous. Hitherto the springs at Ems have been the most celebrated, but Wiesbaden is fast overtaking Ems—in that to its summer attractions it is adding those of winter—it is in fact becoming the winter resort of Germany. Thus while the hotels at Ems are closed for the season and the springs boarded over, Wiesbaden is filled with guests, and bathing and drinking are scarcely abated. The guests this year have numbered almost 100,000,—the city itself has a population of about 50,000. Wiesbaden owes this good fortune to its favorable location. To the north and west it is surrounded by the high hills of the Launees range, and to some extent upon the east, so that the only exposure is a southern one. As to the springs themselves they are almost legion—thirty-two in number, varying in temperature from 50°–155° F.

Dr. Salisbury must have had the Wiesbaden Kochbrunnen in mind when he invented that great American craze—the hot-water cure. Surely extremes do meet when our ice-water dyspeptics undertake to cure themselves with boiling water.

In this famous spring, Kochbrunnen, Nature not only boils but salts the broth—for the water really tastes like broth and is not at all disagreeable, if taken hot. The yield from this one source alone is 17 cubic ft. per minute, while the entire yield of the 32 springs is estimated at 61 cubic ft., and this has continued certainly since the time of Cæsar's invasion, and how much longer man knoweth not, for the origin of the hot springs, according to geology, antedates the origin of man.

For the benefit of those who may wish to recommend these waters to their patients, I enclose their analysis. A pound of Kochbrunnen water contains:

| | | | |
|---|---|---|---|
| Chloride | of Sodium | 52.+ | grs. |
| " | " Potassium | 1.+ | " |
| " | " Lithium | 0.00138 | " |
| " | " Ammonium | 0.12841 | " |
| " | " Calcium | 3.+ | |
| " | " Magnesium | 1.+ | |
| Bromide | " Magnesium | 0.02760 | |
| Iodide | " " | traces | |
| Sulphate | " Lime | 0.69289 | |
| Phosphate | " " | 0.00299 | |
| Arseniate | " " | 0.00115 | |
| Carbonate | " Baryta | traces | |
| " | " Strontia | " | |
| " | " Lime | 3.+ | |

| | | |
|---|---|---|
| " " Magnesia | 0.07979 | |
| " " Protoxide of iron | 0.04339 | |
| " " Manganese | 0.00435 | |
| " " Protoxide copper | traces | |
| Silica | 0.46018 | |
| Silicate of Alumina | 0.00392 | |
| Organic matter | traces | |
| Total solids | 63.+ | grs. |
| Carbonic acid gas | 6.416 | cub. in. |
| Nitrogen | 0.103 | " " |

It is unnecessary to give the analysis of the other springs, save to say that the mineral constituents vary not in quality but in quantity. While the chief springs of Karlsbad and Ems differ only 1-100 from each other in their mineral constituents, those of Wiesbaden differ by 1-4.

The diseases for which these water are recommended are almost innumerable. However, it seems to have become pretty well established that Wiesbaden waters cure rheumatism, while those of Ems cure catarrhal inflammations. How this distinction can be maintained when the waters vary so little in their chemical constituents it is hard to see. It makes one more and more convinced that the efficiency of mineral waters lies not so much in the "minerals" as in the mode of life enforced upon the patient. And this brings us to the consideration of methods. As in everything else in Germany, music forms a part of the programme. At 6 o'clock in the morning, during the spring and summer, the "band begins to play," and the patients begin to drink and promenade, and those who cannot walk are wheeled about in chairs. To this end there are several roofed colonnades leading to the main spring and most beautiful gardens in the immediate vicinity.

Thus the thoughts of the chronic invalid are beguiled, and he begins his day cheerfully, which as we all know is half the battle. The music and drinking continue from 6 o'clock to 8. The water is slowly sipped while the patient is sauntering to and fro, and as you can imagine, it is very hard for a hurrying Chicagoan to assume the true Wiesbaden "saunter and sip." The almost insensible tendency of the "rowdy Westerner" is to leave nothing but the glass at a single draught, and trot off as fast as he came.

The water thus slowly taken into an empty stomach, while the mind is diverted and the lungs receiving fresh air, has a fine chance to wash out the obstructed secretory organs. The morning draught consists of from one to three glasses of ordinary size. This warm and almost neutral saline water cannot help having a beneficial effect upon the mucous membrane of the digestive tract. If the water is taken barely tepid, it acts as a mild aperient, while the hot water has the contrary effect. These draughts are repeated at noon and again at 5 o'clock in the afternoon, so that fresh air, exercise and cheerfulness find a valuable adjuvant in the springs of Germany! The facilities for bathing are really most excellent, though so far I have not been able to find the attendance, which we consider essential to our bathing at home. It seems to me that massage and shampooing would be especially valuable adjuncts to the bath for the rheumatics and paralytics that crowd the shrines here.

The bath rooms are large, light and well-ventilated, the tubs are of marble or stone, and arranged with sprays and douches of all sorts. They are heated by steam in the winter season. Patients are advised not to remain in the water longer than 15 to 20 minutes, not to take the bath hotter than 92° F., and always to rest after the bath, while formerly the patients were allowed to soak for hours in the hottest water. Here, as everywhere, one hour and a half or two hours must be allowed between a meal and a bath. In accordance with human weakness, much more has been claimed for these springs than they are capable of fulfilling—for example, that the worst case of rheumatism is cured in three weeks—the too credulous sufferer finds that three weeks are lengthened into as many months, if not years. Beside mineral baths, Wiesbaden abounds in all sorts of "Caves," such as "grape," "milk," "whey," "fir-needle," etc. I have had an opportunity to see the "grape-cave" in all its glory, as the vintage of the Rhine is just finished.

The grape-cure establishment consists of a fine apartment upon one of the colonnades, where the most delicious grapes are furnished at the smallest prices.

In order to combine the water and grape-cure one can breakfast on grapes in the colonnade after sipping the water, allowing sufficient time for the absorption of the water before taking the grapes.

The regulation amount for breakfast is from one to two pounds, according to the capacity of the person.

For an Englishman who has grown gouty on roast beef and port wine this is surely a fine *régime.*

If our patient felt the need of a more stimulating diet, he might indulge, at suitable intervals, in a glass of "whey," which is brought fresh from the goats every morning.

Really one does feel quite safe here on the milk question. It is one of the "occasions" in which a paternal government is felt to be the best government that man can make for himself. For example, along comes not a milk cart but a woman—women are found to be the cheapest machines everywhere in this country—this woman carries a large can of milk on her head and one in each hand—along comes a government inspector, with the requisite number of bands on his hat and coat—carrying a lactometer. If the milk is found wanting, he turns it into the street and passes on. I should like to send you a few of these uncompromising inspectors to turn over the milk-carts of Chicago,—but here I have really thought the man might carry the cans and allow the woman to carry the lactometer!

The Fir-needle (Kiefernadel) seems to be put to a variety of curative uses. The fir-needle baths are recommended for gout, rheumatism and neuralgia. I have not yet investigated the method.

Then there is a forest-wool (Waldwoll) made from the needles of fir and pine and manufactured into under garments by one Schmidt in the Thuringian forests. The fabric has a golden yellow color and an agreeable pungent odor of pine. A wadding is made of this vegetable wool which is highly recommended by Niemyer and others for bandaging painful joints, etc. As the pungent odor is gradually lost by wear and washing of the fabric, Herr Schmidt has prepared an ethereal oil of this maritime pine, as he calls it, which may be poured or sprinkled upon the under clothes after it has lost its original odor. As the balsamic odor of pine has long been a vaunted remedy for pulmonary catarrhs, why may not this forest-wool underwear be a good compromise for a journey to the pine woods? Possibly this Birnam woods *may* come to Dunsinane. I am reminded, however, that the Birnam woods were oaks instead of pines. Apropos of underwear, the German army is turning its ponderous attention to the subject. Professor Dr. Jäeger, of Stuttgart, recommends an elastic webbing woven from uncolored wool into a single garment, thereby avoiding bands about the waist—a consummation devoutly to be wished.

By the way, the use of Cocain as a local anæsthetic, of which

I wrote you, is becoming quite extensive. Dr. Koller, of the Vienna General Hospital, has been operating successfully with it upon the eye, and its place in the future certainly looks promising. But, unless you have already heard of it, I have to tell you concerning the most remarkable of remedies, viz.: garlic for hydrophobia. The attention of the profession was called to it by an article in a veterinary journal stating the cure of a French peasant. During his ravings he was shut up in a room in which were hanging quantities of garlic roots. He devoured a quantity sufficient to narcotize him and was completely cured. And now it seems that an old Spanish physician—one Victoria Reveira Diaz—possibly a relative of our Dr. Dyas, has been using garlic successfully for some time as a remedy for hydrophobia.

He reports the cure of nine cases in 1882. He applies the remedy internally and externally. The wound is washed and then bound up with powdered garlic. The patient eats two garlic roots with bread every day and swallows sixty grammes of a decoction made from a root of garlic in 720 grammes of water reduced to 500. This treatment lasts for eight days, when the patient is reported cured, but nothing is said as to how long the perfume remains. My attention has often been called to the fact that garlic is a powerful nervine, and it does not seem at all improbable that it might control the fearful spasms of hydrophobia. We shall see.

The latest sensation in the German medical world is the attempt of Bismarck to force an unknown, and, it would seem, unworthy party upon the Faculty of the University at Berlin. This doctor, whose name I have forgotten, cured Bismarck of his obesity and his indigestion, whereupon, by way of gratitude, the Prince must needs call this country doctor to the metropolis and give him a place among the great doctors who had failed to perform the cure. The unknown one from the country called upon his Honor Dubois Reymond and left his cards for both the Professor and his wife. Thereupon the great Dubois returned his cards. A challenge for a duel on the part of the country doctor was the next step in the affair. The challenge was not accepted by Reymond. I am thus reminded of the appointments of the Cook County Commissioners to places of medical distinction, while the quarrels and jealousies and gossip would be "just like a lot of women" were it not for the fact that the parties are all learned professors of the sterner sex. There is a deal of human nature abroad in Germany.

S. H. S.

Nov. 4, 1884.

