# Errata

**Published:** 1885-02

**Authors:** 


ERRATA.

We desire to call attention to the following typographical errors, occurring in the "Letter from Wiesbaden, Germany," appearing in the columns of our last number. On page 30, read "Taunus range" for "Launees range"; on page 33, read "irresistible" for "insensible," "springs" for "shrines"; on page 34, read "cures" for "caves," "regimen" for "*régime*"; on page 35, read "possibly thus" for "possibly this"; on page 37, read "du Bois-Reymond" for "Dubois Reymond."

